# Process Parameters Optimization and Numerical Simulation of AlCoCrFeNi High-Entropy Alloy Coating via Laser Cladding

**DOI:** 10.3390/ma17174243

**Published:** 2024-08-27

**Authors:** Bin Chen, Yang Zhao, Hui Yang, Jingjing Zhao

**Affiliations:** 1 School of Materials Science and Engineering, Anhui University of Technology, Maanshan 243032, China; 2 School of Management Science and Engineering, Anhui University of Technology, Maanshan 243032, China; zhaoyang163yx@163.com (Y.Z.); yanghui07116666@163.com (H.Y.); zjj585939790@163.com (J.Z.)

**Keywords:** laser cladding, AlCoCrFeNi, gray correlation, multi-objective optimization, numerical simulation

## Abstract

The use of laser cladding technology to prepare coatings of AlCoCrFeNi high-entropy alloy holds enormous potential for application. However, the cladding quality will have a considerable effect on the properties of the coatings. In this study, considering the complex coupling relationship between cladding quality and the process parameters, an orthogonal experimental design was employed, with laser power, scanning speed, and powder feed rate as correlation factor variables, and microhardness, dilution rate, and aspect ratio as characteristic variables. The experimental data underwent gray correlation analysis to determine the effect of various process parameters on the quality of cladding. Then, the NSGA-II algorithm was used to establish a multi-objective optimization model of process parameters. Finally, the ANSYS Workbench simulation model was employed to conduct numerical simulations on a group of optimized process parameters and analyze the change rule of the temperature field. The results demonstrate that the laser cladding coating of AlCoCrFeNi high-entropy alloy with the single pass is of high quality within the determined orthogonal experimental parameters. The powder feed rate exerts the most significant influence on microhardness, while laser power has the greatest impact on dilution rate, and scanning speed predominantly affects aspect ratio. The designed third-order polynomial nonlinear regression model exhibits a high fitting accuracy, and the NSGA-II algorithm can be used for multi-objective optimization to obtain the Pareto front solution set. The numerical simulation results demonstrate that the temperature field of AlCoCrFeNi high-entropy alloy laser cladding exhibits a “comet tail” phenomenon, where the highest temperature of the molten pool is close to 3000 °C. The temperature variations in the molten pool align with the features of laser cladding technology. This study lays the groundwork for the widespread application of laser cladding AlCoCrFeNi high-entropy alloy in surface engineering, additive manufacturing, and remanufacturing. Researchers and engineering practitioners can utilize the findings from this research to judiciously manage processing parameters based on the results of gray correlation analysis. Furthermore, the outcomes of multi-objective optimization can assist in the selection of appropriate process parameters aligned with specific application requirements. Additionally, the methodological approach adopted in this study offers valuable insights applicable to the exploration of various materials and diverse additive manufacturing techniques.

## 1. Introduction

High-entropy alloys (HEAs) are alloys composed of five or more elements, with their respective proportions ranging between 5 and 35 atomic percent (at%) [1]. High-entropy alloys have undergone nearly three decades of development since they were first proposed in 1995 [2]. Related studies have demonstrated that compared with traditional alloy materials, HEAs have the characteristics of high entropy phenomenon, cocktail effect, serious lattice distortion, and dynamic hysteresis diffusion [3]. Consequently, high-entropy alloy coatings exhibit superior performance characteristics, including exceptional tensile strength [4], outstanding hardness [5], excellent wear resistance, superior heat resistance, and corrosion resistance [6]. High-entropy alloy powders are predominantly synthesized using techniques such as mechanical alloying [7] and gas atomization [8]. Common preparation methods for high-entropy alloy coatings include vacuum arc melting [9], vacuum induction melting [10], pressureless sintering [11], vacuum hot press sintering [12], magnetron sputtering [13], thermal spraying [14], electrical deposition [15], plasma cladding [16], laser cladding [17], etc. Among these techniques, laser cladding is particularly prevalent due to its advantages of small dilution rate, fast heating and cooling speed, few defects in the cladding layer [18], and good combination effect of substrate and high-entropy alloy [19].

One of the key elements influencing the effect of laser cladding is the processing process parameters. Marzban et al. [20] conducted an orthogonal laser cladding experiment on the surface of AISI 1040 steel and optimized the processing parameters through principal component analysis and approximate ideal solution ordering. Their findings revealed that the laser power exerts the most significant influence on the quality of the cladding. Cui et al. [21] utilized laser cladding technology to fabricate cobalt-based alloy coatings on ZG45 plates, established a regression prediction model of process parameters and evaluation indexes based on experimental results, and analyzed the main influencing factors of cladding quality through the response surface method. In a similar vein, Li et al. [22] investigated the best process variables for laser cladding of WC-Ni60A coating on AISI 304 steel. This was achieved through the comprehensive use of variance analysis, signal-to-noise ratio analysis, and gray correlation analysis. In light of the rapid advancement of computer and artificial intelligence technology, intelligent optimization algorithms have also been integrated into the study of laser cladding-related technology. He et al. [23] constructed a random forest regression prediction model for process parameters and coating effect. They utilized the GNSGA-II algorithm to achieve the Pareto solution set, leading to the selection of the optimal process parameters. In addition, genetic algorithm [24], machine learning [25], neural network [26], particle swarm algorithm [27], etc., are also used to optimize the process parameters of laser cladding. It was found that the evaluation indicators of laser cladding effect should include not only size morphology and dilution rate but also mechanical performance indicators. At the same time, the main processing parameters that affect the laser cladding effect are laser power, scanning speed, and powder feeding rate.

Among many high-entropy alloy systems, AlCoCrFeNi high-entropy alloy has excellent ductility and high-temperature strength and can be used as a protective coating in a special service environment with broad application prospects. Currently, research on AlCoCrFeNi high-entropy alloys is primarily focused on two areas. On the one hand, is the aim is to study the ratio of the five elements. For example, Vo et al. [28] compared and analyzed the performance differences and causes of the two high-entropy alloys Al_0.8_CrFeNi_2.2_ and AlCoCrFeNi_2.1_; Izadi et al. [29] studied the changes in the tissue structure of Al*_x_*CoCrFeNi high-entropy alloys and their influence on corrosion properties under different Al element content; Sim et al. [30] discussed the influence of Ni element content on the characteristics of AlCoCrFeNi*_x_* high-entropy alloys and compared it with AlCoCrFe medium entropy alloys. On the other hand, the research focuses on adding Cu, Mn, Ti [31], Co [32], carbon nanotubes (CNT) [33], TiB_2_ [34], nano TiC [35], Bi_2_O_3_ [36], WC [37], and other trace components to regulate the microstructure, mechanical characteristics, tribological properties, and corrosion resistance of AlCoCrFeNi high-entropy alloys. However, the optimization of process parameters for laser cladding of AlCoCrFeNi high-entropy alloys is not perfect, and there is an immediate requirement to develop a forecasting model and a multi-objective optimization model for the correlation between the process parameters and the geometrical quality of the coatings as well as the mechanical properties.

The objectives of this study are to investigate the effects of different processing parameters (laser power, scanning speed, and powder feed rate) on the quality of laser cladding AlCoCrFeNi high-entropy alloy coatings (microhardness, dilution rate, and aspect ratio), to develop empirical models correlating these parameters with cladding quality, and to design a multi-objective optimization method for process parameters. This study aims to establish a foundation for the extensive application of laser cladding AlCoCrFeNi high-entropy alloys in surface engineering, additive manufacturing, and remanufacturing, while providing insights relevant to process studies across various materials and additive manufacturing technologies. The research approach adopted in this study involves the following: orthogonal experiments were initially conducted on single-pass laser cladding of AlCoCrFeNi high-entropy alloy to investigate the variations in cladding quality under different laser cladding process parameters. Subsequently, the data from the orthogonal experiments were then analyzed to assess the impact of various process parameters on the cladding quality evaluation indexes using the gray correlation analysis method. Furthermore, a multivariate nonlinear regression model was constructed through polynomial fitting of laser cladding process parameters and various cladding quality evaluation indexes. Based on the aforementioned findings, a multi-objective optimization design of the process parameters was carried out using the NSGA-II algorithm. Finally, a numerical simulation was performed using the finite element model in ANSYS Workbench for a set of the optimized process parameters, followed by an analysis of the variations in the temperature field. Compared to previous studies, the research framework of this paper is more comprehensive, encompassing a broader range of evaluation criteria for clad quality. The employed research methods are more precise and stable, while effectively mitigating the time and economic costs associated with extensive experimentation.

## 2. Materials and Methods

### 2.1. Experimental Design

H13 steel is an ideal choice for manufacturing molds and tools under high-strength and high-temperature working conditions due to its excellent thermal, mechanical, and processing properties. It can be undergo surface cladding for enhanced properties before use, or subjected to additive repair after prolonged use. Therefore, we have chosen the widely used H13 steel as the experimental substrate, measuring 60 mm × 60 mm × 10 mm, with its material chemical composition detailed in Table 1. The cladding powder utilized was AlCoCrFeNi high-entropy alloy powder, with a diameter range of 15~53 μm, and its chemical composition is presented in Table 2.

**Figure 1:**
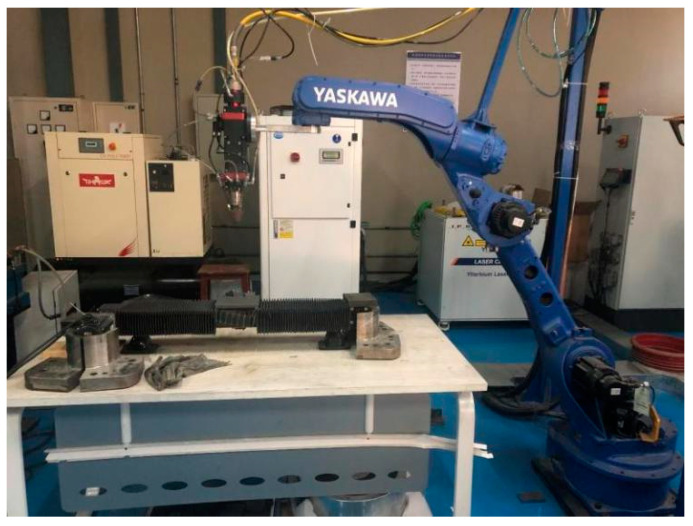
YLS-4000-KC coaxial powder feeding laser cladding equipment.

The AlCoCrFeNi high-entropy alloy coating was fabricated on the surface of H13 steel using the YLS-4000-KC coaxial powder feeding laser cladding equipment (Nanjing Zhongke Yucheng Laser Technology Co., Ltd., Nanjing, China), as illustrated in Figure 1. Prior to cladding, the surface of the H13 substrate was polished and cleaned. This was followed by descaling with P400-P1000 sandpaper until the surface was smooth and clean. The surface was then cleaned using anhydrous ethanol and subsequently air-dried, rendering it ready for use. The laser cladding spot diameter was 3 mm, the protective gas was high-purity argon, the gas pressure was 0.5 MPa, and the carrier gas flow rate was 12 dm^3^/min. According to existing literature, combined with the exploration of preliminary experiments and practical engineering applications, the experiment was primarily focused on the control of three process parameters: laser power *P* (kW), scanning speed *v* (mm/s), and powder feeding rate *q* (r/min). Each parameter had four levels, and a 3-factor, 4-level L16 (43) orthogonal experimental plan was employed, as shown in Table 3. Among them, the laser powers used were 2.1 kW, 2.2 kW, 2.3 kW, and 2.4 kW, respectively. The scanning speeds were 8 mm/s, 10 mm/s, 12 mm/s, and 14 mm/s, respectively. The powder delivery rates were 0.6 r/min (~9.76 g/min), 0.8 r/min (~11.89 g/min), 1.0 r/min (~14.03 g/min), and 1.2 r/min (~16.24 g/min), respectively.

**Figure 2:**
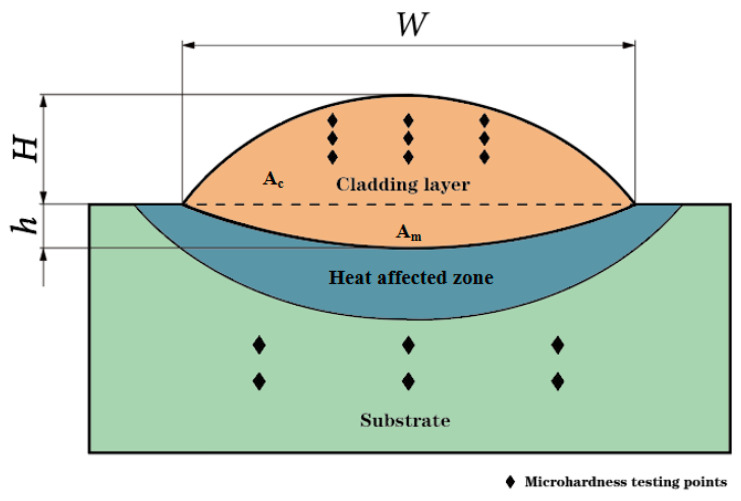
Schematic of laser cladding cross-section measurement.

Laser cladding technology has strong stability, and the geometric shape changes of different cross-sections are relatively small. Therefore, this article selects the most representative middle-position cross-section for characterization. The cross-section of the AlCoCrFeNi high-entropy alloy coating was prepared by cutting, grinding, and polishing. Ultrasonic cleaning was employed to remove surface contamination, and corrosion was induced using aqua regia for a duration of 1 min. The width *W* (mm), height *H* (mm), and depth of the fusion zone *h* (mm) of the fusion cladding of a layer of AlCoCrFeNi high-entropy alloy were observed and measured under the microscope. The HVS-1000 microhardness measuring instrument was employed to ascertain the microhardness of both the substrate and the fusion cladding layer. The indenter utilized was a hardened steel ball with a pressure of 1 kg. In order to assure the accuracy of the microhardness measurements, the substrate (H13) was measured six times, with the maximum and minimum values removed and the average taken as the microhardness of the substrate. On the AlCoCrFeNi high-entropy alloy coating cross-section, we selected nine measurement points to remove the maximum and minimum values, and the average value was used to calculate the microhardness of the cladding layer. The method is shown in Figure 2.

### 2.2. Gray Correlation Theory

In order to determine the optimal process parameters, namely laser power *P*, scanning speed *v*, and powder feeding rate *q*, for the laser cladding of AlCoCrFeNi high-entropy alloy, microhardness *HV*, dilution rate *η*, and aspect ratio *W*/*H* were selected as the evaluation criteria for the cladding quality. The dilution rate *η* can be represented using the melting area of the substrate (A_m_) and the cladding area (A_c_), which is calculated as Am/(Ac + Am), or simplified as *h*/(*H* + *h*). Regions A_c_ and A_m_ are shown in Figure 2. Gray correlation theory is a robust approach for analyzing the intrinsic correlation between uncertainty and ambiguity data, offering high accuracy and stability in data processing [38]. Therefore, this theory was employed to investigate how each process parameter affected cladding quality.

The laser power *P*, scanning speed *v*, and powder feeding rate *q*, which are the parameters of the laser cladding process, are defined as correlation factor variables, denoted as *x_i_* (*i* = 1, 2, 3). Conversely, the microhardness *HV*, dilution rate *η*, and aspect ratio *W*/*H*, which are the indicators of cladding quality, are defined as characteristic variables, denoted as *y_j_* (*j* = 1, 2, 3). If orthogonal experiments were conducted in a total of *n* groups, the sequence of correlation factors *X_i_* (*x_i_*(1), *x_i_*(2), *x_i_*(*k*), ···, *x_i_*(*n*)) and the sequence of features *Y_j_* (*y_j_*(1), *y_j_*(2), ···, *y_j_*(*k*), ···, *y_j_*(*n*)) would be constituted. Given the considerable disparity in the magnitude and order of magnitude of individual parameters in the evaluation indexes of processing process parameters and cladding quality, it is imperative to normalize the sequences of correlation factors *X_i_* and the sequences of features *Y_j_* through the homogenization operator *D_AVG_*, thereby obtaining $X_{i}^{AVG}$ and $Y_{j}^{AVG}$, as shown in Equations (1) and (2).

(1) $$X_{i}^{AVG}=X_{i}D_{AVG}=(x_{i}^{AVG}(1),x_{i}^{AVG}(2),\cdots ,x_{i}^{AVG}(k),\cdots x_{i}^{AVG}(n))$$

(2) $$Y_{j}^{AVG}=Y_{j}D_{AVG}=(y_{j}^{AVG}(1),y_{j}^{AVG}(2),\cdots ,y_{j}^{AVG}(k),\cdots y_{j}^{AVG}(n))$$

Among them, $x_{i}^{AVG}$(*k*) and $y_{j}^{AVG}$(*k*) can be calculated using Equations (3) and (4), respectively.

(3) $$x_{i}^{AVG}(k)=x_{i}(k)/(\frac{1}{n}\sum_{k=1}^{n}x_{i}(k))$$

(4) $$y_{j}^{AVG}(k)=y_{j}(k)/(\frac{1}{n}\sum_{k=1}^{n}y_{j}(k))$$

The absolute difference matrix Δ*_ji_*(*k*) between the normalized sequence of correlation factor $X_{i}^{AVG}$ and the normalized sequence of feature $Y_{j}^{AVG}$ is calculated according to Equation (5).

(5) $$\Delta_{ji}(k)=\left|y_{j}^{AVG}(k)-x_{i}^{AVG}(k)\right|$$

The gray correlation coefficient is a measure of the geometric distance between the sequence of correlation factors and the sequence of features at each sample point. As the value increases, the correlation becomes stronger. The gray correlation coefficient *γ_ji_*(*k*) is calculated using Equation (6).

(6) $$\gamma_{ji}(k)=\frac{\underset{i}{\min}\;\underset{k}{\min}\;\Delta_{ji}(k)+\xi \;\underset{i}{\max}\;\underset{k}{\max}\;\Delta_{ji}(k)}{\Delta_{ji}(k)+\xi \;\underset{i}{\max}\;\underset{k}{\max}\;\Delta_{ji}(k)}$$

where the constant *ξ* is employed to mitigate the impact of excessive Δ*_ji_*(*k*) on the gray correlation calculation and to rectify the distortion caused by bias. The excessive Δ*_ji_*(*k*) may be attributed to the extreme value and uncertainty of the orthogonal experiments of laser cladding for AlCoCrFeNi high-entropy alloy. In most cases, *ξ* is set within the range of 0~1.

The gray correlation coefficient *γ_ji_*(*y_j_*(*k*), *x_i_*(*k*)) is used to express the degree of correlation between different sample points. In cases where the distribution of values is more dispersed, it is necessary to integrate the method of using the mean value of multiple gray correlation coefficients. In order to reflect the degree of correlation between the sequence of correlation factors and the sequence of features from the overall, the larger the value, the stronger the correlation, and the comprehensive gray correlation coefficient *ϕ_ji_* is calculated by Equation (7).

(7) $$\phi_{ji}=\frac{1}{n}\sum_{k=1}^{n}\gamma_{ji}(k)$$

### 2.3. Multi-Objective Optimization Model

**Figure 3:**
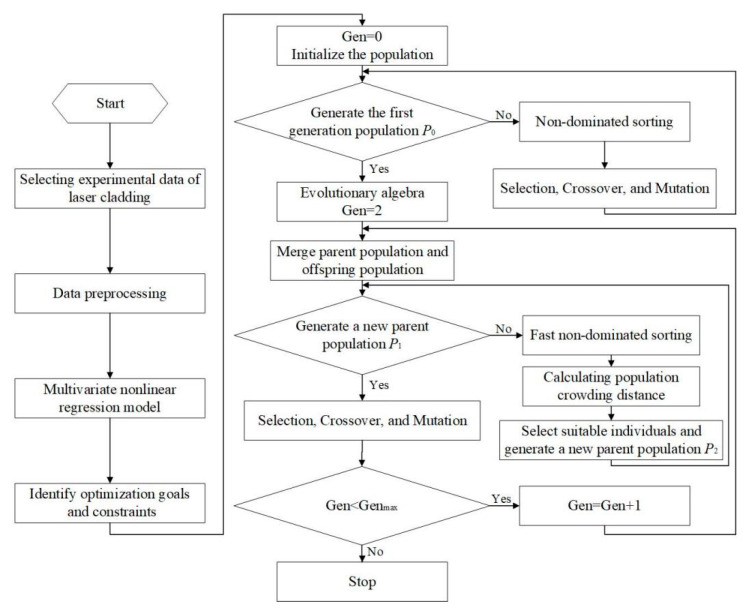
Multi-objective optimization workflow for process parameters.

The multi-objective optimization process of the laser cladding process for AlCoCrFeNi high-entropy alloy is illustrated in Figure 3. The multivariate nonlinear regression model of the laser cladding process parameters and each cladding quality evaluation index are constructed separately, with the NSGA-II algorithm used for the multi-objective optimization. The NSGA-II algorithm employs a three-step process to maintain the diversity of the populations: nondominated sorting, congestion computation, and elite strategy. This approach helps the algorithm rapidly converge to the globally optimal solution while avoiding the emergence of local optima [39].

In actual production and application, the AlCoCrFeNi high-entropy alloy fused cladding layer must exhibit high strength and good wear resistance. Therefore, the greater the microhardness *HV*, the better [40]. A dilution rate of *η* too small will result in a reduction of the bonding zone between the fused cladding layer and the H13 substrate, making it challenging to achieve a metallurgical bond with optimal performance. Conversely, a dilution rate of *η* too large will lead to a reduction in powder utilization, increasing the likelihood of cracking and deformation in the fused cladding layer. Therefore, this paper sets a limit on the dilution rate of *η* between 30% and 50% [41]. When the aspect ratio *W*/*H* is too small, it will affect the wetting performance of the cladding layer and also have an unfavorable impact on subsequent multi-pass cladding processes, leading to a decrease in cladding efficiency. Therefore, a larger aspect ratio *W*/*H* is preferable [42]. Drawing from the aforementioned analysis, considering providing decision-makers with a wider range of references and avoiding situations such as non-optimal solutions, the multi-objective optimization model for laser cladding of AlCoCrFeNi high-entropy alloy can be obtained as Equation (8).

(8) $$\left\{\begin{array}{l} HV=f_{1\max}\left(P,\;v,\;q\right)\\ \eta =f_{2}\left(P,\;v,\;q\right)\in \left[0.3,\;0.5\right]\\ W/H=f_{3\max}\left(P,\;v,\;q\right) \end{array}\right.$$

where *f*_1_, *f*_2_, and *f*_3_ are used to indicate the multivariate nonlinear regression models between the laser cladding process parameters and the dependent variables microhardness *HV*, dilution rate *η*, and aspect ratio *W*/*H*, respectively.

The constraint conditions are the ranges of various processing parameters in the laser cladding experiment of AlCoCrFeNi high-entropy alloy, as shown in Equation (9).

(9) $$s.t.\;\left\{\begin{array}{l} 2.1\leq P\leq 2.4\\ 8\leq v\leq 14\\ 9.76\leq q\leq 16.24 \end{array}\right.$$

## 3. Results and Discussion

### 3.1. Orthogonal Experiment Results

**Figure 4:**
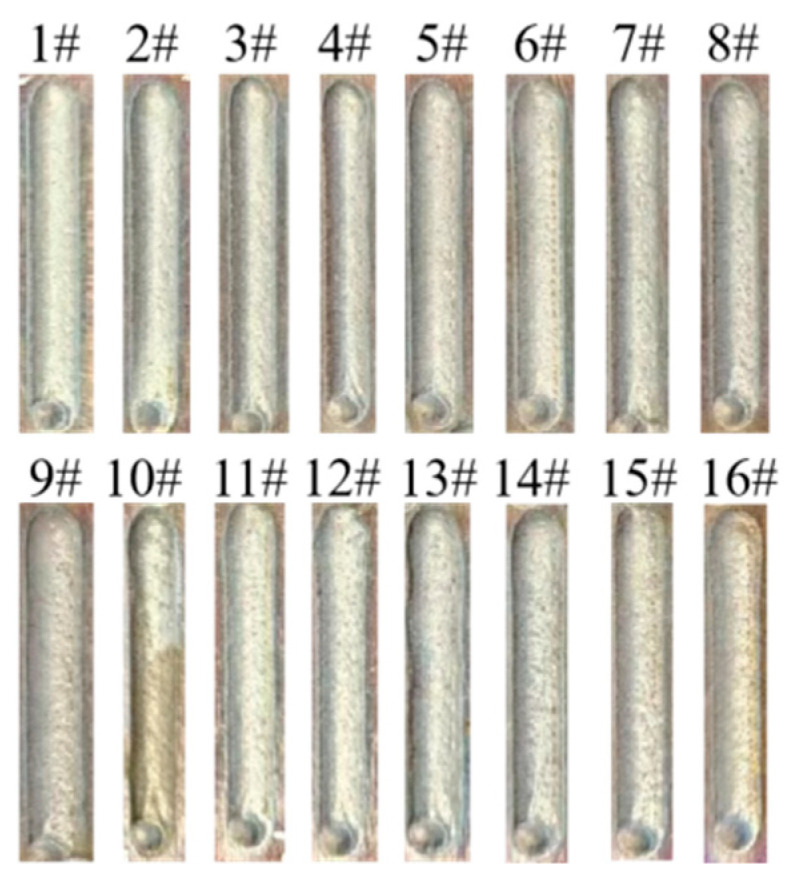
Sixteen groups of single passes morphology.

**Figure 5:**
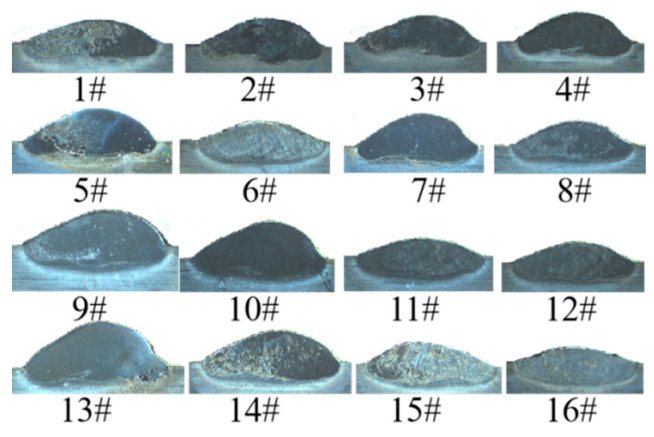
Cross-sectional morphology of the cladding layer.

Figure 4 depicts the surface morphology of 16 groups of single passes of the AlCoCrFeNi high-entropy alloy laser cladding orthogonal experiment. It can be observed that the macroscopic quality of the cladding is satisfactory, the surface roughness is minimal, and there are no discernible defects such as cracks, folds, and so forth. The cross-sectional morphology is depicted in Figure 5, demonstrating that the fusion cladding layer renders a strong metallurgical link with the substrate, and no defects such as cracks and peeling are observed. The orthogonal experimental scheme and cross-sectional measurement results are shown in Table 4. From the data in Table 4, it can be seen that during the laser cladding process of AlCoCrFeNi high-entropy alloy, the mechanism by which process parameters affect the cladding effect is very complex, and no obvious change law can be found from the data alone.

### 3.2. Gray Correlation Analysis

According to Equations (1)–(4) and Table 4, the 16 sets of orthogonal experimental data for laser cladding of AlCoCrFeNi high-entropy alloy were normalized, resulting in the normalized sequences of relevant factors $X_{i}^{AVG}$ and the normalized sequences of characteristic $Y_{j}^{AVG}$, as shown in Table 5.

Based on Equation (5) and Table 5, the absolute difference matrix Δ*_ji_*(*k*) between the normalized sequences of relevant factors $X_{i}^{AVG}$ and the normalized sequences of characteristic $Y_{j}^{AVG}$ was calculated, as shown in Table 6.

*ξ* can be adjusted for the actual system. Based on previous research and the requirements of this study, equal weight responses can be selected. Therefore, parameter *ξ* is taken as 0.5 [43,44,45,46]. According to the data in Table 6 and Equation (6), the gray correlation coefficient *γ_ji_*(*k*) was calculated, as shown in Table 7.

The comprehensive gray correlation coefficient *ϕ_ji_* was calculated in accordance with Table 7 and Equation (7), as illustrated in Table 8. It can be seen from the table that the comprehensive gray correlation coefficient between laser power, scanning speed, powder feeding rate, and microhardness are 0.640, 0.564, and 0.744, respectively. The powder feeding rate has the greatest impact on microhardness, followed by laser power and scanning speed. The variation of these three parameters, especially the powder feeding rate, leads to different amounts of AlCoCrFeNi high-entropy alloy powder melting and ultimately results in changes in microhardness.

The comprehensive gray correlation coefficient between laser power, scanning speed, powder feeding rate, and dilution rate are 0.742, 0.730, and 0.590, respectively. Laser power has the greatest impact on the dilution rate, followed by scanning speed and powder feeding rate. The AlCoCrFeNi high-entropy alloy powder absorbs the majority of the laser energy during laser cladding, with the residual energy reaching the substrate and causing it to melt and form a molten pool. Therefore, changes in laser energy lead to different volumes of the molten pool, which in turn cause variations in the dilution rate. Scanning speed and powder feeding rate affect the volume of high-entropy alloy powder penetrated by the laser within a unit of time. Different degrees of laser energy attenuation lead to changes in the dilution rate.

The comprehensive gray correlation coefficient between laser power, scanning speed, powder feeding rate, and the aspect ratio are 0.686, 0.706, and 0.579, respectively. The most significant factor affecting the aspect ratio is scanning speed, which is followed by laser power and powder feeding rate. Scanning speed and powder feeding rate primarily affect the amount of AlCoCrFeNi high-entropy alloy powder melted per unit time, which in turn influences the width and height of the cladding layer. Laser power affects the melting and fluidity of the powder, which, under the effects of gravity and Marangoni convection, further impacts the morphology of the cladding layer.

Analogous phenomena and patterns have been identified by other scholars in their investigation of the GS-Fe01 iron-based alloy powder laser cladding process [47]. In conclusion, in the actual AlCoCrFeNi high-entropy alloy laser melting and cladding production and application, it is of paramount importance to prioritize the control of the powder feeding rate if the objective is to achieve a higher microhardness, to prioritize the control of the laser power if the objective is to achieve a lower dilution rate, and to prioritize the control of the scanning speed if the objective is to achieve larger aspect ratio.

### 3.3. Multiple Nonlinear Regression Results

**Figure 6:**
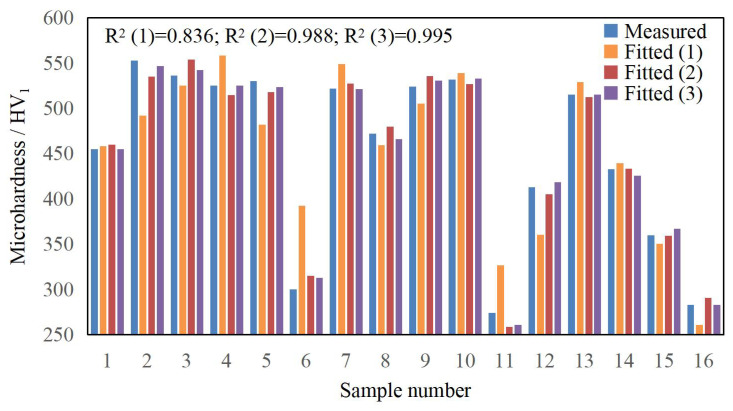
Microhardness fitting bar graphs.

**Figure 7:**
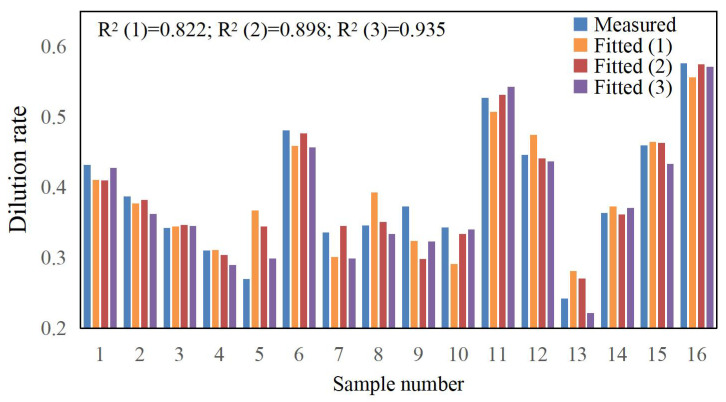
Dilution rate fitting bar graphs.

**Figure 8:**
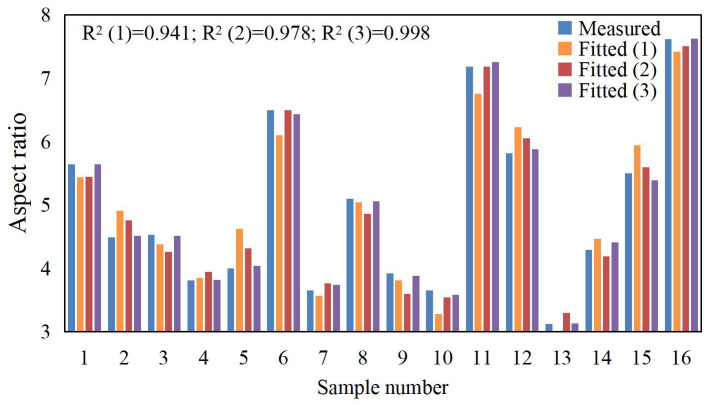
Aspect ratio fitting bar graphs.

According to the data in Table 3, polynomial fitting was performed for the laser cladding process parameters and various cladding quality evaluation indicators. During the actual fitting process, it was found that the first-order polynomial fit was poor, the second-order polynomial fit was good, and introducing a third-order polynomial improved the fitting accuracy, as shown in Figure 6, Figure 7 and Figure 8. The fitting function is given by Equation (10).

(10) $$f=a_{0}+a_{1}P+a_{2}v+a_{3}q+a_{4}P^{2}+a_{5}v^{2}+a_{6}q^{2}++a_{7}Pv+a_{8}Pq+a_{9}vq+a_{10}P^{3}+a_{11}v^{3}+a_{12}q^{3}$$

**Figure 9:**
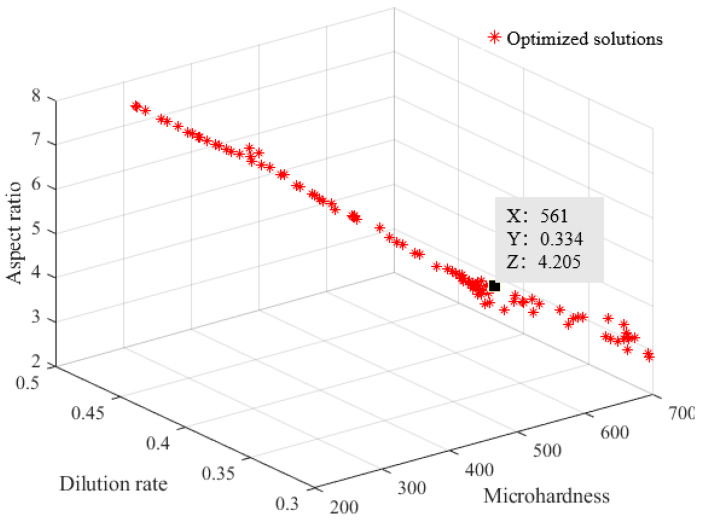
The optimized solution set of the Pareto front.

The fitting functions for microhardness *HV*, dilution rate *η*, and aspect ratio *W*/*H* are denoted as *f*_1_, *f*_2_, and *f*_3_, respectively. The experimental data from Table 4 were input into Equation (10) and processed using Matlab (R2017b) software to compute the correlation coefficient (*a*_0_, *a*_1_, *a*_2_, …, *a*_12_). In order to fully display the polynomial fitting results and ensure calculation accuracy, the complete numerical values of the coefficients in the corresponding Equation (10) are shown in Table 9, and the fitting bar graphs are depicted in Figure 7, Figure 8 and Figure 9. From the figures, it can be seen that the correlation coefficient R² for the microhardness fitting function *f*_1_ is 0.995, for the dilution rate fitting function *f*_2_, it is 0.935, and for the aspect ratio fitting function *f*_3_, it is 0.998. The correlation coefficients for all three fitting functions are close to 1, and there are no outliers, indicating high fitting and prediction accuracy.

### 3.4. Multi-Objective Optimization Results

The NSGA-II algorithm was employed for the multi-objective optimization of the process parameters for AlCoCrFeNi high-entropy alloy laser cladding. The fitting function in Section 3.3 was incorporated into the multi-objective optimization model (8), with a population size set to 100, maximum iteration set to 300, crossover probability set to 0.8, and mutation probability set to 0.05. The optimized solution set of the Pareto front is shown in Figure 9. The figure shows that the Pareto front solution set of this optimization contains 97 points. These solutions are all feasible solutions of the optimization objective under the constraints. The solution to multi-objective optimization problems is not unique but a set of Pareto optimal solutions. These Pareto optimal or non-dominated optimal solutions represent the best trade-offs or compromises that can be achieved between objectives. It can be clearly seen from the graph that selecting appropriate design variable values in order to obtain a better target value will result in the deterioration of other target values, and no point can simultaneously meet all three target requirements. In the actual production and application, one set of processing parameters can be selected based on the specific service conditions and requirements of AlCoCrFeNi high-entropy alloy laser cladding.

The optimization, in this instance, selected a set of results with lower dilution rate, better aspect ratio, and higher hardness. These were rounded according to the actual operating requirements of the laser cladding equipment. The corresponding laser cladding process parameters are laser power *P* = 2.129 kW, scanning speed *v* = 10 mm/s, and powder feeding rate *q* = 0.9 r/min (~12.98 g/min). The optimal process parameters for laser cladding of AlCoCrFeNi high-entropy alloy were experimentally verified, and the results are shown in Table 10. The final measured microhardness was *HV* = 560 HV_1_, the dilution rate was *η* = 0.345, and the aspect ratio was *W*/*H* = 4.208. Compared to the predicted results, the deviations were minimal, at 2.68%, 6.09%, and 1.56%, respectively.

### 3.5. Numerical Simulation

#### 3.5.1. Theoretical Basis

The laser cladding of AlCoCrFeNi high-entropy alloy involves a series of complex physicochemical processes, and it is difficult to obtain the temperature distribution in the molten pool through experiments. Therefore, it is necessary to characterize its variation law through finite element numerical simulation methods. However, existing numerical simulation tools find it challenging to consider all factors of the cladding process. To improve the numerical simulation accuracy and computational efficiency, it is necessary to simplify and make assumptions about the laser cladding numerical model [24,48]:

(1) Assume that, except for density, coefficient of thermal expansion, thermal conductivity, specific heat capacity, Young’s modulus, and Poisson’s ratio, which change with temperature, the thermal physical parameters of AlCoCrFeNi high-entropy alloy powder and H13 substrate do not change with temperature;
(2) Assume that the powder and the substrate are both isotropic and uniformly continuous, and ignore the influence of cracks, voids, and other defects inside the substrate and cladding layer on the material performance parameters;
(3) Assume that the laser power is constant and ignore the influence of gases in the air on the laser power;
(4) Ignore the influence of powder velocity, flow rate, and pressure of high-purity argon gas used as shielding gas, as well as the chemical reactions, liquid flow, and stirring effect inside the molten pool, on the temperature;
(5) Neglect the heat exchange between the H13 substrate and the laser cladding experimental platform;
(6) Neglect the influence of interface thermal resistance at the cladding layer–substrate interface, solid–liquid interface, and other interfaces on heat transfer.

The heat transfer process of laser cladding for AlCoCrFeNi high-entropy alloy can be expressed as Equation (11).

(11) $$\rho C\frac{\partial T}{\partial t}=\frac{\partial }{\partial x}\left(k_{x}\frac{\partial T}{\partial x}\right)+\frac{\partial }{\partial y}\left(k_{y}\frac{\partial T}{\partial y}\right)+\frac{\partial }{\partial z}\left(k_{z}\frac{\partial T}{\partial z}\right)+Q$$

where *x*, *y*, and *z* are the Cartesian coordinate system directions of the model, *ρ* is the density of the material, *C* is the specific heat capacity of the material, *T* is the temperature field distribution function, *t* is the time, *Q* is the internal heat source, *k_x_*, *k_y_*, and *k_z_* are the heat transfer coefficients in the *x*, *y*, and *z* directions, respectively, and, since the material is isotropic and assumed to be homogeneous and continuous, *k_x_* =*k_y_* =*k_z_* [49].

In order to calculate and solve the laser cladding temperature field of AlCoCrFeNi high-entropy alloy, it is required to calculate the starting temperature distribution and boundary conditions according to the experiment environment. The initial conditions are set to Equation (12).

(12) $$T(x,\;y,\;z,\;t=0)=T_{0}$$

*T*_0_ is the starting temperature.

Considering the presence of heat conduction, thermal convection, and thermal radiation in the cladding process, the boundary conditions are set to Equation (13).

(13) $$k_{x}\frac{\partial T}{\partial x}n_{x}+k_{y}\frac{\partial T}{\partial y}n_{y}+k_{z}\frac{\partial T}{\partial z}n_{z}=\left\{\begin{array}{l} T_{s}\left(x,\;y,\;z,\;t\right)\\ q_{s}\left(x,\;y,\;z,\;t\right)\\ h\left(T_{\alpha }-T_{s}\right) \end{array}\right.$$

where *T_s_* and *T_α_* are the boundary temperature and ambient temperature, *q_s_* is the density of heat flow from the heat source to the surface, *h* is the composite heat transfer coefficient, and *n_x_*, *n_y_*, and *n_z_* are the cosine values in the direction normal to the outside of the boundary [50].

The energy for the melting of AlCoCrFeNi high-entropy alloy powder comes from the spot energy emitted by the laser, and existing studies show that the heat flow density of the spot has a normal Gaussian distribution [51]. In order to demonstrate the pinhole effect during laser cladding, we used a Gaussian distributed cone heat source; the model for the heat source can be formulated as Equation (14).

(14) $$q\left(x,\;y,\;z\right)=\frac{9P}{\pi hR_{0}^{2}(1-e^{-3})}\exp\left(-\frac{9({\left(x-vt\right)}^{2}+y^{2})}{R_{0}^{2}\log(h/z)}\right)$$

where *q*(*x*, *y*, *z*) is the heat flow density at any point, *P* is the laser power at the time of the experiment, *R*_0_ is the radius of the laser spot at the time of the experiment, *v* is the laser spot moving speed, and *h* is the effective depth of heat flow.

#### 3.5.2. Construction of Finite Element Models

**Figure 10:**
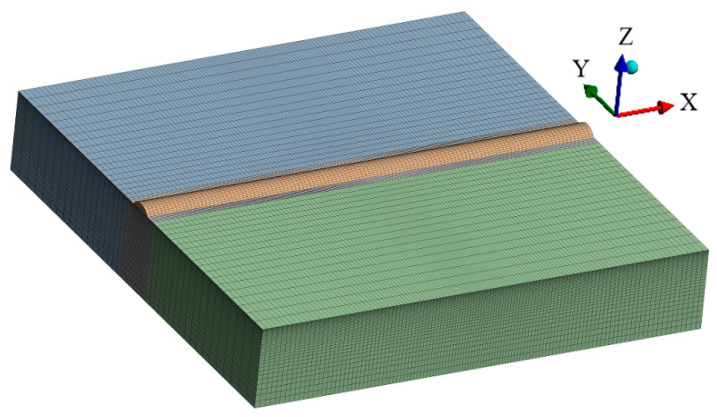
Three-dimensional simulation model.

**Figure 11:**
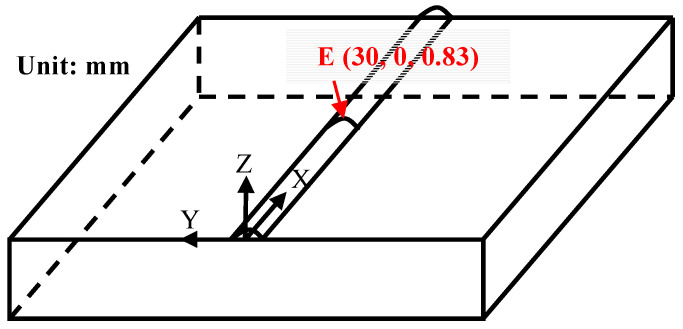
Important nodes and paths in the Model.

A three-dimensional simulation model of AlCoCrFeNi high-entropy alloy laser cladding was established using ANSYS Workbench finite element 24.1 software for numerical simulation. The substrate size is 60 mm × 60 mm × 10 mm, and the cladding layer is obtained by stretching a circular arc with a height of 0.83 mm and a width of 3.43 mm over a length of 60 mm, as shown in Figure 10. In order to ensure calculation accuracy and save computational time while improving efficiency, a refined mesh was applied in the cladding layer and nearby regions, while a relatively coarser mesh was used in areas far from the cladding layer, adopting a gradual transition approach. The cladding layer and substrate are both made of hexahedral mesh. The arc surface of the cladding layer is divided into 16 elements, the arc width direction is divided into 14 elements, and the cladding length direction is divided into 120 elements. The X, Y, and Z directions of the substrate contain 120, 58, and 34 elements, respectively. The final model consisted of 240,480 elements and 1,001,839 nodes, with the temperature field element type being Solid279. To conduct a comprehensive analysis of the temperature field in the laser cladding process, critical nodes and important cross-sections of the model were selected for further analysis, as shown in Figure 11.

The laser cladding process parameters utilize the optimized parameters from Section 3.4, namely laser power *P* = 2.129 kW, scanning speed *v* = 10 mm/s, laser spot diameter *d* = 3 mm, and powder feeding rate *q* = 0.9 r/min (~12.98 g/min). The density, thermal conductivity, specific heat, and thermal expansion coefficient of the material were calculated using JMatPro 7.0 software. The calculated thermal physical properties of the substrate H13 steel and AlCoCrFeNi high-entropy alloy are listed in Table 11 and Table 12, respectively. The thermal physical properties of the material within the remaining temperature range are obtained through linear interpolation and extrapolation methods. The AlCoCrFeNi high-entropy alloy powder melts at 1550 °C, slightly above the melting point of the substrate H13 steel, which is approximately 1470 °C. The starting temperature of the model is set to 22. The heat input is simulated by a Gaussian distributed conical heat source, as shown in formula (14). Thermal convection uses the convective heat transfer coefficient of air. The degrees of freedom of the bottom nodes of the model are fully constrained.

To realistically simulate coaxial laser cladding, the “birth and death” element technique in ANSYS Workbench was used to achieve real-time filling of the cladding layer. In this numerical simulation, a time step takes 0.05 s. Before applying the heat source, all cladding layer elements were set to the “death” state. When subjected to the heat source, the “dead” elements were reactivated. Thus, as the laser spot scans, the cladding layer is progressively activated, achieving a real-time filling process for the cladding layer.

#### 3.5.3. Temperature Field Analysis

**Figure 12:**
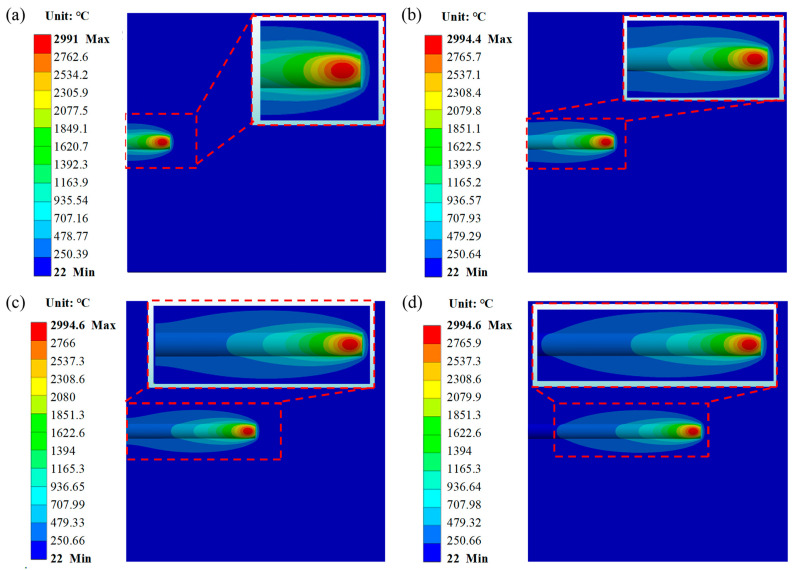
Temperature fields of the model at different times: (**a**) t = 1 s, (**b**) t = 2 s, (**c**) t = 3 s, (**d**) t = 4 s.

The moment at which the laser heat source begins to interact with the H13 steel substrate and the AlCoCrFeNi high-entropy alloy is defined as t = 0. The temperature field distributions of the model at t = 1 s, t = 2 s, t = 3 s, and t = 4 s are shown in Figure 12a–d, respectively, with the highest temperatures being close to 3000 °C, with values of 2991 °C, 2994.4 °C, 2994.6 °C, and 2994.6 °C, respectively. Figure 12 also shows that the temperature field distribution exhibits a “comet tail” phenomenon, which deviates from the ideal normal Gaussian distribution of the laser heat source. In the vicinity of the laser spot, the isotherm is in close proximity to the circle. Conversely, the “comet tail” phenomenon becomes more evident as the distance from the core increases. The main reasons for this phenomenon are that, first, during the coaxial laser cladding process, the laser spot moves linearly along the X-axis, meaning the temperature of the already clad area has not completely cooled. Second, the isotherm density and temperature gradient are high near the center of the laser spot, while the isotherm density and temperature gradient are low farther from the center, making the phenomenon more evident in distant regions. Due to the higher thermal conductivity of the AlCoCrFeNi coating compared to that of the melted powder and even the unmelted powder, and also due to influence by the distance from the heat source and thermal exchange with the surrounding environment, the isotherms are sparser at the tail end of the “comet tail”. The “comet tail” phenomenon indicates that the optimized processing parameters help to reduce the temperature gradient inside the sample, thereby reducing the thermal stress during the formation of the coating [52]. The numerical simulation results also indicate that the “comet tail” formed around and behind the melt pool exhibits a uniform and continuous morphology, without fractures or significant irregularities. The length, width, and density of the “comet tail” are moderate. This indicates that the optimized laser cladding process has a uniform distribution of heat input and heat flow on the material surface, which is conducive to surface flatness and the formation of consistent microstructure and material properties of the AlCoCrFeNi coating. Comparing Figure 12a–d, the differences in the highest temperatures in the model’s temperature field are minor as the laser cladding progresses, and the distribution patterns remain essentially consistent. This indicates that the optimized AlCoCrFeNi high entropy alloy laser cladding process is stable and the selected processing parameters are appropriate.

**Figure 13:**
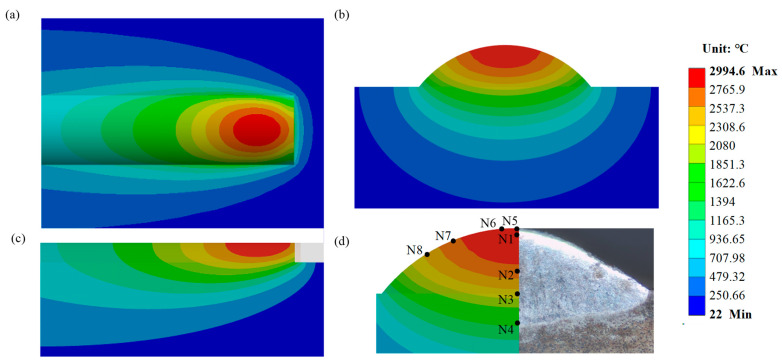
Temperature field at the highest temperature of point E: (**a**) the XY plane projection, (**b**) the YZ cross-section, (**c**) the XZ cross-section, (**d**) comparison of the molten pool morphology between numerical simulation and experiment results.

**Figure 14:**
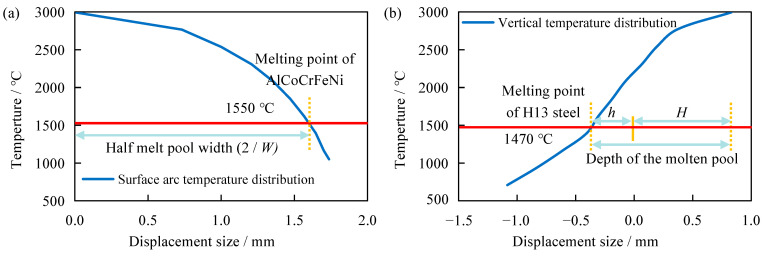
Measurement diagram of molten pool morphology: (**a**) measurement of the width of the molten pool, (**b**) measurement of the depth of the molten pool. The red line in the figure is the temperature scale line, and the temperature value has been marked in the attachment of the red line.

To delve deeper into the distribution patterns of the temperature field during laser cladding for AlCoCrFeNi high-entropy alloy, key nodes and important cross-sections were analyzed. Figure 13 depicts the temperature field distribution of the model at point E when it reaches the highest temperature. Specifically, (a) represents the temperature field distribution in the XY plane projection, (b) represents the temperature field distribution in the YZ cross-section, (c) represents the temperature field distribution in the XZ cross-section, and (d) represents the morphology of the molten pool. From Figure 13a, it can be seen that the XY plane projection temperature field distribution exhibits a distinct “comet tail” phenomenon, consistent with the distribution pattern in Figure 12. The XZ cross–section temperature field distribution in Figure 13c also illustrates the presence of the “comet tail” phenomenon, further demonstrating that the isotherm density and temperature gradient are higher near the center of the laser spot, while they are lower farther from the center, with the effect becoming more pronounced at greater distances. Further analysis of the YZ cross-section temperature field distribution in Figure 13b and the molten pool morphology in Figure 13d reveals that the contour of the molten pool is approximately elliptical. The experimental melt pool morphology is in good agreement with the simulation results, which verifies the rationality of the model. Referring to existing literature, the temperature field distribution of the molten pool obtained through numerical simulation is used to calculate the size of the molten pool and compared with experimental results to verify the accuracy of the finite element model [24,52]. According to Figure 13, the molten pool morphology measurement map has been further drawn, as shown in Figure 14. From the surface arc temperature distribution in Figure 14a and the vertical temperature distribution in Figure 14b, it can be seen that the numerical simulation yielded a cladding width of approximately 3.232 mm, height of approximately 0.830 mm, and depth of approximately 0.373 mm. The simulated dimensions show errors within 10% compared to the experimental measurements of the cladding (width of 3.435 mm, height of 0.829 mm, depth of 0.398 mm), indicating minor deviations. This demonstrates the high precision of the ANSYS Workbench finite element model and the numerical simulation process. Researchers can conduct comparative analyses of other processing parameters based on this foundation. Especially when there are multiple available laser cladding processes to choose from, our approach can help identify more reasonable processing parameters.

**Figure 15:**
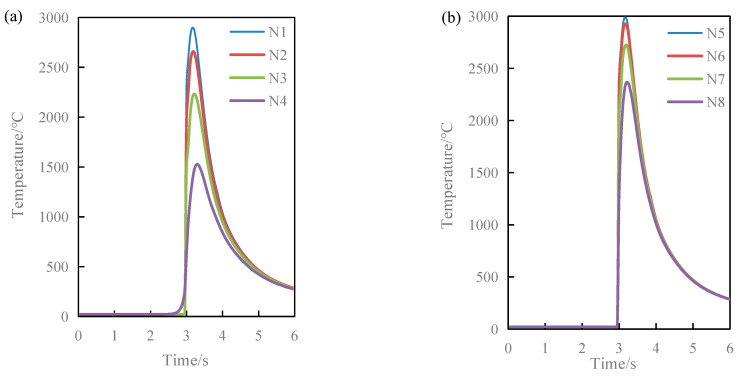
The temperature–time history curves of key nodes: (**a**) nodes N1, N2, N3, and N4 in the vertical direction, (**b**) nodes N5, N6, N7, and N8 in the arc direction.

Figure 15a,b respectively show the temperature–time history curves of nodes N1, N2, N3, and N4 in the vertical direction and nodes N5, N6, N7, and N8 in the arc direction at point E on the YZ cross-section. From Figure 15, it can be seen that the closer the distance to the center of the laser spot, the higher the temperature. For the nodes on the arc, the reason for this phenomenon is that the distance from nodes N5 to N8 to the center of the laser spot is getting farther and farther, and the laser heat source model follows a Gaussian distribution. The energy acting on these nodes decreases sequentially, ultimately resulting in a change in the highest temperature. For the nodes in the vertical direction, the primary explanation for this phenomenon is that the heat irradiated on the material surface by the laser is influenced by comprehensive factors such as thermal conduction during vertical transmission. However, the temperature variation patterns over time are generally consistent across all nodes: the temperature rapidly rises when the laser spot center approaches and quickly drops when the laser spot center moves away. This also verifies the characteristic of rapid heating and cooling in laser cladding. Due to the heat dissipation conditions and the release of latent heat of crystallization during the solidification process of the H13 and AlCoCrFeNi high-entropy alloy in the molten pool, the cooling rate is lower than the heating rate. Additionally, it can also be seen from Figure 15 that the highest temperatures at each node and the duration of temperatures above the melting point of the material are relatively short. This indicates that the optimized process can achieve a more uniform solidification process, reducing the time for grain growth and solute element segregation, which is beneficial for fine-grain strengthening and thereby improves the quality of AlCoCrFeNi high-entropy alloy coating by laser cladding.

## 4. Conclusions

The article comprehensively employs methods such as orthogonal experiments, gray relational analysis, multiple linear regression, multi-objective optimization design based on the NSGA-II algorithm, and finite element numerical simulation based on ANSYS Workbench to analyze the laser cladding process of AlCoCrFeNi high-entropy alloy. Firstly, 16 groups of single-pass AlCoCrFeNi high-entropy alloy coatings were prepared on the surface of H13 steel using YLS-4000-KC coaxial powder feeding laser cladding equipment. The macroscopic quality of the single pass cladding was good, the surface roughness was low, and there were no obvious defects such as cracks and wrinkles, which proves the feasibility of the laser cladding technology of AlCoCrFeNi high-entropy alloys, and it also provides the basis for subsequent research. Secondly, gray correlation analysis of the data obtained from the orthogonal experiments shows that the powder feed rate exerts the most significant influence on microhardness, while laser power has the greatest impact on dilution rate, and scanning speed predominantly affects aspect ratio during the AlCoCrFeNi high-entropy alloy laser cladding process. Thirdly, the third-order polynomial nonlinear regression model of AlCoCrFeNi high-entropy alloy laser cladding process parameters and the cladding quality evaluation indexes has high fitting accuracy, and the Pareto frontier solution set is obtained by multi-objective optimization using the NSGA-II algorithm, which can be used to select the appropriate laser cladding process parameters according to the actual cladding requirements. Finally, numerical simulation results showed that the temperature field of AlCoCrFeNi high-entropy alloy laser cladding exhibited a “comet tail” phenomenon, where the highest temperature in the molten pool is close to 3000 °C. The changes in the molten pool temperature field are consistent with the characteristics of the laser cladding process. We further demonstrated the feasibility of optimizing process technology and the adaptability of laser cladding of AlCoCrFeNi high-entropy alloy powder.

This foundational exploration aims to advance the application of this technology in fields such as surface engineering, additive manufacturing, and remanufacturing repairs. Future research will delve into the multi-pass and multi-layer laser cladding techniques for AlCoCrFeNi high-entropy alloys and their microstructures and properties. Researchers and engineering practitioners can utilize the findings from this research to judiciously manage processing parameters based on the results of gray correlation analysis. The outcomes of multi-objective optimization can assist in the selection of appropriate process parameters aligned with specific application requirements. At the same time, numerical simulation techniques are applied to verify the rationality of optimization results, accurately predict the melting and solidification behavior of powders, reduce experimental costs and time, and thus accelerate the development process. In addition, the paper provides important references for the laser cladding process of other high-entropy alloy materials and offers ideas into the process analysis and optimization of other advanced manufacturing technologies.

## Figures and Tables

**Table 1:** Chemical composition of H13 steel (mass fraction, wt%).

Element	C	Si	Mn	Cr	Mo	V	P	S	Fe
Value	0.39	0.83	0.38	5.00	1.22	0.86	0.022	0.005	Bal.

**Table 2:** Chemical composition of AlCoCrFeNi high-entropy alloy powder (mass fraction, wt%).

Element	Al	Co	Cr	Fe	Ni
Value	10.69	23.34	20.60	22.12	23.25

**Table 3:** Experimental factors and levels of laser cladding for AlCoCrFeNi high-entropy alloy.

Levels	Factors		
	Laser Power *P*/kW	Scanning Speed *v*/mm·s^−1^	Powder Feeding Rate *q*/g·min^−1^
Level 1	2.1	8	9.76
Level 2	2.2	10	11.89
Level 3	2.3	12	14.03
Level 4	2.4	14	16.24

**Table 4:** Orthogonal experimental plan and cross-sectional measurement results.

Number	Process Parameters			Measurement Results		
	Laser Power *P*/kW	Scanning Speed *v*/mm·s^−1^	Powder Feeding Rate q/g·min^−1^	Microhardness *HV*/HV_1_	Dilution Rate *η*	Aspect Ratio *W*/*H*
1#	2.1	8	9.76	455	0.432	5.644
2#	2.1	10	11.89	553	0.387	4.488
3#	2.1	12	14.03	536	0.342	4.531
4#	2.1	14	16.24	525	0.310	3.810
5#	2.2	8	11.89	530	0.270	3.999
6#	2.2	10	9.76	300	0.481	6.493
7#	2.2	12	16.24	522	0.336	3.654
8#	2.2	14	14.03	472	0.346	5.097
9#	2.3	8	14.03	524	0.373	3.918
10#	2.3	10	16.24	532	0.343	3.651
11#	2.3	12	9.76	274	0.527	7.186
12#	2.3	14	11.89	413	0.446	5.821
13#	2.4	8	16.24	515	0.242	3.124
14#	2.4	10	14.03	433	0.364	4.289
15#	2.4	12	11.89	360	0.460	5.498
16#	2.4	14	9.76	283	0.576	7.617

**Table 5:** The normalized sequences of relevant factors $X_{i}^{AVG}$ and the normalized sequences of characteristic $Y_{j}^{AVG}$.

$X_{i}^{AVG}$			$Y_{j}^{AVG}$		
$x_{1}^{AVG}(k)$	$x_{2}^{AVG}(k)$	$x_{3}^{AVG}(k)$	$y_{1}^{AVG}(k)$	$y_{2}^{AVG}(k)$	$y_{3}^{AVG}(k)$
0.933	0.727	0.752	1.007	1.108	1.146
0.933	0.909	0.916	1.224	0.992	0.911
0.933	1.091	1.081	1.187	0.878	0.920
0.933	1.273	1.251	1.162	0.795	0.773
0.978	0.727	0.916	1.173	0.694	0.812
0.978	0.909	0.752	0.664	1.235	1.318
0.978	1.091	1.251	1.156	0.863	0.742
0.978	1.273	1.081	1.045	0.888	1.035
1.022	0.727	1.081	1.160	0.956	0.795
1.022	0.909	1.251	1.178	0.879	0.741
1.022	1.091	0.752	0.607	1.353	1.459
1.022	1.273	0.916	0.914	1.145	1.182
1.067	0.727	1.251	1.140	0.620	0.634
1.067	0.909	1.081	0.959	0.935	0.871
1.067	1.091	0.916	0.797	1.181	1.116
1.067	1.273	0.752	0.627	1.477	1.546

**Table 6:** The normalized sequences of relevant factors $X_{i}^{AVG}$ and the normalized sequences of characteristic $Y_{j}^{AVG}$.

Δ_11_(*k*)	Δ_12_(*k*)	Δ_13_(*k*)	Δ_21_(*k*)	Δ_22_(*k*)	Δ_23_(*k*)	Δ_31_(*k*)	Δ_32_(*k*)	Δ_33_(*k*)
0.074	0.280	0.255	0.175	0.381	0.356	0.212	0.419	0.394
0.291	0.315	0.308	0.059	0.083	0.076	0.022	0.002	0.005
0.253	0.096	0.106	0.055	0.213	0.203	0.014	0.171	0.161
0.229	0.110	0.089	0.138	0.478	0.456	0.160	0.499	0.478
0.196	0.446	0.257	0.284	0.033	0.222	0.166	0.084	0.104
0.314	0.245	0.088	0.257	0.325	0.483	0.340	0.409	0.566
0.178	0.065	0.095	0.114	0.227	0.388	0.236	0.349	0.509
0.067	0.228	0.036	0.090	0.385	0.193	0.057	0.238	0.046
0.138	0.433	0.079	0.066	0.229	0.125	0.227	0.068	0.286
0.156	0.269	0.073	0.143	0.030	0.372	0.281	0.168	0.510
0.416	0.484	0.145	0.331	0.262	0.601	0.437	0.368	0.707
0.108	0.358	0.002	0.122	0.128	0.229	0.159	0.091	0.266
0.074	0.413	0.111	0.447	0.107	0.631	0.432	0.093	0.617
0.108	0.050	0.122	0.132	0.026	0.146	0.196	0.038	0.210
0.270	0.294	0.119	0.115	0.090	0.265	0.050	0.025	0.200
0.440	0.646	0.125	0.410	0.204	0.725	0.480	0.274	0.794

**Table 7:** The gray correlation coefficient *γ_ji_*(*k*).

*γ*_11_(*k*)	*γ*_12_(*k*)	*γ*_13_(*k*)	*γ*_21_(*k*)	*γ*_22_(*k*)	*γ*_23_(*k*)	*γ*_31_(*k*)	*γ*_32_(*k*)	*γ*_33_(*k*)
0.818	0.538	0.561	0.723	0.522	0.540	0.655	0.489	0.504
0.529	0.509	0.514	0.922	0.872	0.886	0.951	1.000	0.992
0.563	0.775	0.757	0.929	0.675	0.687	0.971	0.702	0.715
0.588	0.749	0.788	0.776	0.462	0.474	0.716	0.445	0.456
0.626	0.422	0.560	0.601	0.981	0.664	0.709	0.828	0.796
0.510	0.572	0.790	0.627	0.564	0.460	0.541	0.495	0.414
0.648	0.837	0.776	0.814	0.658	0.518	0.630	0.535	0.440
0.832	0.590	0.905	0.858	0.520	0.699	0.879	0.628	0.900
0.705	0.430	0.807	0.907	0.656	0.797	0.639	0.858	0.584
0.678	0.549	0.819	0.768	0.990	0.529	0.588	0.706	0.440
0.440	0.402	0.693	0.560	0.621	0.403	0.479	0.522	0.361
0.754	0.477	1.000	0.801	0.792	0.657	0.717	0.817	0.602
0.819	0.441	0.748	0.480	0.827	0.391	0.481	0.814	0.394
0.753	0.872	0.729	0.786	1.000	0.764	0.673	0.916	0.657
0.548	0.526	0.735	0.814	0.858	0.619	0.893	0.945	0.668
0.426	0.335	0.724	0.502	0.685	0.357	0.455	0.595	0.335

**Table 8:** The comprehensive gray correlation coefficient *ϕ_ji_*.

*ϕ_ji_*	Laser Power *P*	Scanning Speed *v*	Powder Feeding Rate *q*
Microhardness *HV*	0.640	0.564	0.744
Dilution rate *η*	0.742	0.730	0.590
Aspect ratio *W*/*H*	0.686	0.706	0.579

**Table 9:** Values of the coefficients in the fitted function.

Coefficient	Fit Function		
	*f*_1_	*f*_2_	*f*_3_
*a*_0_	60,753.53995	293.88582	726.92022
*a*_1_	−81,476.78321	−387.21358	−882.57317
*a*_2_	−190.68071	0.18548	−0.18753
*a*_3_	811.55869	−1.06937	−15.93261
*a*_4_	34,936.43423	172.31339	397.49202
*a*_5_	−6.29712	−0.02943	0.07772
*a*_6_	−51.06455	0.08443	1.14057
*a*_7_	111.25000	0.07909	−0.02923
*a*_8_	−4.93924	−0.03661	0.19536
*a*_9_	−3.29580	5.77689 × 10^−4^	−0.01491
*a*_10_	−5089.65692	−25.53765	−59.64352
*a*_11_	0.28883	7.74861 × 10^−4^	−0.00271
*a*_12_	1.16376	−0.00208	−0.02800

**Table 10:** Multi-objective optimization model prediction results and experimental results.

	Microhardness *HV*/*HV*_1_	Dilution Rate *η*	Aspect Ratio *W*/*H*
Prediction results	561	0.334	4.205
Experimental results	575	0.324	4.142
Error/%	2.50	2.99	1.50

**Table 11:** The calculated thermal physical properties of H13.

Temperature (°C)	Density (g/(cm)^3^)	Specific Heat (J/(g K))	Thermal Conductivity (W/m K)	Thermal Expansion Coefficient (10^−6^/K)
22	7.74232	0.44793	34.22998	12.20303
200	7.69096	0.53212	33.8028	12.35203
400	7.62551	0.64751	32.08765	13.44289
600	7.55277	0.84721	29.19734	14.43505
800	7.47502	0.84593	27.48423	15.29497
1000	7.45094	0.71905	28.05636	13.30179
1400	7.20363	1.96216	32.34514	18.07875
1800	6.69282	0.83024	37.11	29.40634
2200	6.34178	0.83247	43.76672	31.59778
2600	5.9743	0.83321	50.42344	38.27681
3000	5.60184	0.83343	57.08016	42.78304

**Table 12:** The calculated thermal physical properties of AlCoCrFeNi.

Temperature (°C)	Density (g/(cm)^3^)	Specific Heat (J/(g K))	Thermal Conductivity (W/m K)	Thermal Expansion Coefficient (10^−6^/K)
22	7.05515	0.50448	42.66598	13.80349
200	6.97521	0.60063	42.35551	21.0365
400	6.90243	0.64244	31.54551	19.29267
600	6.81968	0.71864	32.81245	19.7689
800	6.71872	0.84129	35.08276	21.35085
1000	6.58944	0.86145	38.85223	24.01175
1400	6.02251	0.78449	28.49336	41.44993
1800	5.70202	0.83381	33.83801	44.46904
2200	5.36094	0.83714	39.18266	48.35054
2600	5.01208	0.83714	44.52732	52.69254
3000	4.66577	0.83714	49.87197	57.3093

## Data Availability

The authors declare that the data supporting the findings of this study are available within the article.
